# Circular RNA hsa_circ_0008003 facilitates tumorigenesis and development of non‐small cell lung carcinoma via modulating miR‐488/ZNF281 axis

**DOI:** 10.1111/jcmm.15987

**Published:** 2020-12-15

**Authors:** Runhuan Gu, Koufeng Shao, Qiaoxia Xu, Xue Zhao, Haibing Qiu, Haibo Hu

**Affiliations:** ^1^ Department of Oncology The Affiliated Huai’an Hospital of Xuzhou Medical University The Second People’s Hospital of Huai’an Huai’an China; ^2^ Department of Oncology Huai’an Chuzhou Hospital of Traditional Chinese Medicine Zhongda Hospital Group Hospital Addiliated to Southest University Huai’an China; ^3^ Nursing Department Huaiyin Hospital of Huai’an City Huai’an China; ^4^ Department of Thoracic Surgery The Affiliated Huai’an Hospital of Xuzhou Medical University The Second People’s Hospital of Huai’an Huai’an China; ^5^ Department of Respiratory Huaiyin Hospital of Huai’an City Huai’an China

**Keywords:** ceRNA, circRNA, miR‐488, non‐small cell lung cancer, ZNF281

## Abstract

As one of the most aggressive malignancies, non‐small cell lung carcinoma (NSCLC) has high risks of death. It has been demonstrated that circRNAs accelerate NSCLC progression, but the underlying molecular mechanisms of circRNAs in NSCLC were still obscure. In the first place, the circRNA microarray of NSCLC was investigated in this study, and *hsa_circ_0008003* (*circ‐0008003*) was chosen as the research object. Then, it was unveiled that the expression of *circ‐0008003* examined via qRT‐PCR was elevated in tumour tissues relative to the non‐tumour tissues, which was associated with TNM stage and lymphatic metastasis in NSCLC. Additionally, the prognosis of NSCLC patients with high *circ‐0008003* level was poor. Besides, *circ‐0008003* silencing dampened the invasion and proliferation of NSCLC cells. Next, according to the mechanistic studies, *circ‐0008003* functioned as a ceRNA of *ZNF281* in NSCLC by acting as the endogenous sponge for miR‐488, which was proved to be a tumour suppressor in NSCLC. Additionally, *ZNF281* overexpression and miR‐488 suppression recovered the influences of repressed *circ‐0008003* on NSCLC cellular processes. It was validated in this research that *circ‐0008003* triggered tumour formation in NSCLC, which was adjusted via miR‐488/*ZNF281* axis, casting a novel light on the resultful target for treating NSCLC and predicting the prognosis.

## 1 INTRODUCTION

Non‐small cell lung cancer (NSCLC) is a major cause of death associated with lung cancer, taking up over 85% of all lung cancers.^1^ The survival of patients can be prolonged by diagnosing and treating the disease in the early stage. Nevertheless, the potential mechanism by which lung cancer evolves remains undocumented. Advances made in RNA research recently have contributed to identification of non‐coding RNA (ncRNA) implicated in different biological processes.^2^, ^3^ LncRNAs and miRNAs are verified in previous research to function in the evolution of lung cancer, especially in adjustment of apoptosis, proliferation and invasion.^4^, ^5^, ^6^, ^7^ Herein, ncRNAs need to be researched to continuously confirm the mechanism associated with the cancer from the aspect of epigenetics.

As a newfound type of RNAs, circular RNAs (circRNAs) display a wide expression in eukaryotes. As bioinformatics and high‐flux sequencing have boomed in recent years, our sight on circRNAs has been notably broadened. Most circRNAs reported recently are non‐coding, but some of them encode proteins or polypeptides according to reports.^8^, ^9^ Through non‐canonical splicing, circRNAs generate with relative resistance to degradation by exonucleases.^10^, ^11^

Inextricable associations are found between circRNAs and the development of multiple disorders, especially that of cancer.^12^, ^13^ As circRNAs have gradually been extensively analysed, their functions as tumour oncogenes or suppressors are identified through adjusting the apoptosis, migration, proliferation, invasion and metastasis of tumour cells.^14^, ^15^, ^16^, ^17^ For instance, circRNA *hsa_circ_0001649* inhibits HCC progression via multiple miRNA sponge^18^; circRNA 0001785 functions as a competing endogenous RNA (ceRNA) to adjust the mechanism of osteosarcoma by sponging miR‐1200 so as to elevate HOXB2^19^; *hsa_circ_0091570* acts as a ceRNA to impede the evolution of HCC by sponging hsa‐miR‐1307.^20^ However, the effect of circRNAs in NSCLC needs continuous explorations.

In the current research, the expression of *circ‐0008003* in NSCLC was discussed, and the function and potential regulatory mechanism of *circ‐0008003* were then investigated. The findings unfolded that *circ‐0008003* served as a ceRNA to mediate *ZNF281* by sponging miR‐488 in NSCLC.

## 2 MATERIALS AND METHODS

### 2.1 Tissue samples

Thirty NSCLC tissues and 30 matched para‐cancerous tissues were collected from NSCLC cases subjected to excision in The Affiliated Huai'an Hospital of Xuzhou Medical University and The Second People's Hospital of Huai'an. Immediately after collection, liquid nitrogen was used to freeze all the samples prior to the extraction of total RNA. All participants signed the informed consent for scientific investigations. This research was conducted in line with the Helsinki Declaration, and it was monitored and approved by the Ethics Committee of The Affiliated Huai'an Hospital of Xuzhou Medical University and The Second People's Hospital of Huai'an.

### 2.2 Cell lines and transfection

The Cell Bank of Chinese Academy of Sciences provided five human NSCLC cell lines (CAL‐12T, HCC827, A549, H460 and H1299) and one human bronchial epithelioid cell line (16HBE). They were cultured in RPMI 1640 medium (HyClone) with 1% penicillin/streptomycin (Gibco) and 10% foetal bovine serum (FBS; Pan Life Sciences) and maintained under a humid circumstance with 5% CO_2_ at 37°C.

Subsequently, 1 × 10^6^ recombinant lentivirus‐transducing units and 6 μg/mL Polybrene (Sigma) were utilized to infect cells in the case of the fusion of 60%‐80%. Treatment with 2 μg/mL puromycin was adopted for the selection of cells with 2 weeks of stable transfection, and these cells were chosen through flow cytometry for later experiments. GenePharma provided lentiviruses and plasmids applied in this research.

### 2.3 Quantitative reverse transcription‐polymerase chain reaction (qRT‐PCR)

In line with the regimen of the manufacturer, the total RNA extraction from tissues and cells was performed using TRIzol reagent (Invitrogen), followed by qRT‐PCR mentioned above.^21^ Specific primers used in this research were shown below:

GAPDH: forward: 5′‐TGACTTCAACAGCGACACCCA‐3′,

reverse: 5′‐CACCCTGTTGCTGTAGCCAAA‐3′;

miR‐488: forward: 5′‐ACACTCCAGCTGGGTTGAAAGGCTATTTC‐3′,

reverse: 5′‐CTCAACTGGTGTCGTGGAGTCGGCAATTCAG

TTGAGGACCAAGA ‐3′

circ‐0008003: forward: 5′‐TGGGGCTAACAAACTTCACC‐3′,

reverse: 5′‐GTCCCACCAGAGGTTGTCAC‐3′.

### 2.4 Cell proliferation assay

Based on the regimen of the manufacturer, cell proliferation was examined via Cell Counting Kit‐8 (CCK8) (Beyotime) assay. Following 24 hours of culture in a 96‐well plate, the cells were subjected to 1 hour of incubation with CCK8 in an incubator for cell culture. A TECAN infinite M200 Multimode microplate reader (TECAN) was employed to test the cell optical density at 450 nm.

For EDU assay, EDU labelled solution (KeyGen Biotech) was used. Nucleus staining was performed by adding DAPI.

### 2.5 Cell invasion assay

By reference to the regimen of the manufacturer, the invasion capacity of cells was assessed by Transwell assay. Transwell chambers (Millipore Corporation) pre‐coated with matrigel were then used. During invasion assessment, the upper chamber was inoculated with 2 × 10^5^ cells in medium with no serum, whereas the lower chamber was added with 600 μL of medium with 10% FBS as a chemoattractant. Following 24 hours of incubation, cells subjected to migration were fixed and dyed with a 5% crystal violet solution (Beyotime), and the images of cells undergoing migration were captured by an optical microscope (Olympus Corporation). Moreover, Image‐pro Plus 6.0 (Media Cybernetics) was utilized to count the migrated cells.

### 2.6 Luciferase reporter assay

Wild‐type plasmids *circ‐0008003*‐WT and *ZNF281*‐WT containing binding sites for miR‐488 and mutant plasmids *circ‐0008003*‐MUT and *ZNF281*‐MUT were consolidated into the pGL3 promoter vector (GenePharma). After the seeding of NSCLC cells into a 24‐well plate, the cells underwent cotransfection with 80 ng plasmids + 5 ng pRL‐SV40 and 50 nmol/L miR‐488 mimics or negative control (NC) with the help of Lipofectamine 2000 reagent. After that, the luciferase activity was assessed using the dual‐luciferase reporter assay kit (Promega). All experiments were carried out thrice, respectively.

### 2.7 RNA reduction assay

RNAs were labelled with SP6/T7 RNA polymerase (Roche) and the Biotin RNA labelling mix (Roche Diagnostics) in vitro and treated with RNase‐free DNase I (Roche). Then, RNeasy Mini Kit (Qiagen) was utilized for the purification of RNAs labelled with biotin (Bio‐miR‐488‐Mut, Bio‐miR‐488‐WT and Bio‐NC). Thereafter, the mixed solution (50 pmol of biotinylated RNA and 1 mg NSCLC cell) was added with washed beads (6 mL) containing streptavidin agarose (Life Technologies), and 1 hour of incubation was carried out at room temperature. After the rinsing of beads, the amplification of eluted RNAs was conducted, followed by measurement of them via qRT‐PCR.

### 2.8 RNA immunoprecipitation (RIP) assay

By reference to the guidance of the manufacturer, RIP assay was carried out with the use of the Magna RIP kit (Millipore). Specifically, lysis buffer (Gibco) containing RNase inhibitor was used to lyse H460 and H1299 cells. After that, protein A/G agarose beads with AGO2 antibody were added to incubate cell lysates, with normal rabbit IgG as NC. Then, the RNAs that were co‐precipitated underwent washing, purification and examination using qRT‐PCR.

### 2.9 RNA‐Fluorescence In Situ Hybridization (RNA‐FISH)

RNA‐FISH was conducted using *circ‐0008003* probes conjugated with fluorescence by reference to the former research.^22^ Then, DNA probe sets (Biosearch Technologies) were employed for hybridization based on the regimen of Biosearch Technologies. Ultimately, a FV1000 confocal laser microscope (Olympus) was utilized for probing cells.

### 2.10 In vivo assays

Shanghai SLAC Laboratory Animal Co. Ltd. provided female nude mice aged 4 weeks, which were randomly allocated into two groups: 1 × 10^7^ *circ‐0008003* shRNA H460 cells in 100 μL of medium with no serum were injected at the left flank in one group (n = 4), while control cells treated with NC were injected in the other group (n = 4). Afterwards, a calliper was applied to test and calculate the tumour size every other day on the basis of the formula: size = length×width^2^/2. Prior to tumour excision and photography, the mice were killed after 3 weeks. Then immunohistochemistry (IHC) staining was carried out with the fixed tumours. Based on the Care and Use of Laboratory Animals of the National Institutes of Health, assays were conducted.

### 2.11 Immunohistochemistry staining

Ki‐67 antibody and PCNA antibody (Abcam) were used for IHC staining by reference to the regimen mentioned above.^23^

### 2.12 Statistical analysis

Experimental data (obtained from experiments conducted thrice) were expressed as the mean ± standard deviation (SD). Intergroup comparison was carried out using the Student's *t* test, and Kaplan‐Meier (K‐M) curves were plotted for the comparison of overall survival via log‐rank test. To figure out the correlation between clinicopathological characteristics and *circ‐0008003* expression, Fisher's exact test or chi‐square test was employed. Besides, miR‐488's association with *circ‐0008003*/*ZNF281* expression was determined by Pearson's correlation analysis. *P* < .05 meant a statistical significance.

## 3 RESULTS

### 3.1 Abnormal *circ‐0008003* expression was observed in NSCLC tissues and cells

**Figure 1:**
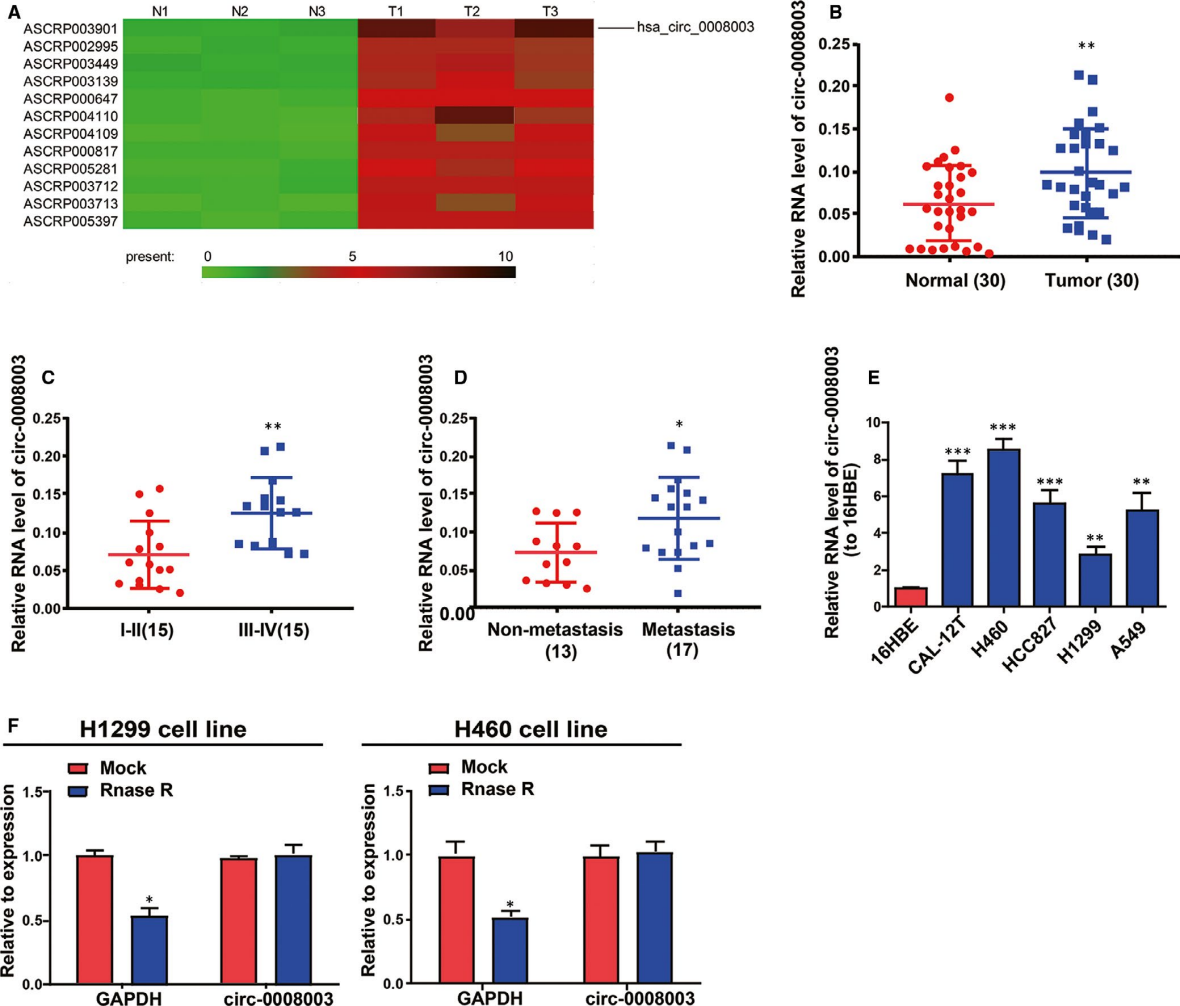
*Circ‐0008003* is raised in NSCLC tissues and cells. GSE112214 (platform GPL19978) microarray analysis is conducted for three NSCLC samples and three matched adjacent normal samples. On the basis of the criteria of *P* < .05 and fold change >4, the differential expression of circRNAs including *circ‐0008003* is confirmed in NSCLC tissues. The expression levels of *circ‐0008003* in human NSCLC tissues and cell lines are detected by qRT‐PCR. A, Heat map shows differentially expressed circRNAs with statistically significance between NSCLC samples and three matched adjacent normal samples. *Circ‐0008003* expression is elevated in NSCLC samples. B, Up‐regulation of *circ‐0008003* in NSCLC tissues (n = 30) compared with matched para‐carcinoma tissues (n = 30). C, Expression of *circ‐0008003* in high TNM stage (n = 15) is higher than in low TNM stage (n = 15). D, The level of *circ‐0008003* is raised in tissues undergoing lymphatic metastasis (n = 17) compared with those undergoing no lymphatic metastasis (n = 13). E, *Circ‐0008003* expression in NSCLC cell lines (CAL‐12T, H460, HCC827, H1299 and A549) and the human bronchial epithelioid cell line 16HBE. F, Resistance of *circ‐0008003* in NSCLC cells to RNase R digestion. Data are the mean ± SEM. *P < .05, **P < .01, ***P < .001

GSE112214 microarray analysis demonstrated that *circ‐0008003* displayed a high expression in NSCLC tissues. On the basis of the criteria of *P* < .05 and fold change >4, the different expression of circRNAs including *circ‐0008003* was confirmed in NSCLC tissues (Figure 1A). To verify the role of *circ‐0008003* in NSCLC, qRT‐PCR was adopted to test its expression in NSCLC tissues and matched non‐tumour tissues. According to the findings, NSCLC tissues had remarkably raised *circ‐0008003* expression relative to non‐tumour tissues (Figure 1B). And *circ‐0008003* level in TNM stage Ⅰ‐Ⅱ was evidently lower than that in TNM stage Ⅲ‐Ⅳ (Figure 1C). Besides, tissues undergoing lymphatic metastasis had higher *circ‐0008003* expression than those undergoing no lymphatic metastasis (Figure 1D). Additionally, *circ‐0008003* relative expression in NSCLC cells was evidently raised relative to human bronchial epithelioid cell line 16HBE (Figure 1E). Because of the highest or lowest *circ‐0008003* expressions in H460 and H1299 cells, they were picked for later assays.^24^, ^25^ *Circ‐0008003* has resistance to RNase R digestion, so it was considered to possess circRNA features (Figure 1F). These results suggest the elevated aberrant expression of *circ‐0008003* and its role in NSCLC as an oncogene.

### 3.2 The promotive effect of *circ‐0008003* on NSCLC cellular processes

**Figure 2:**
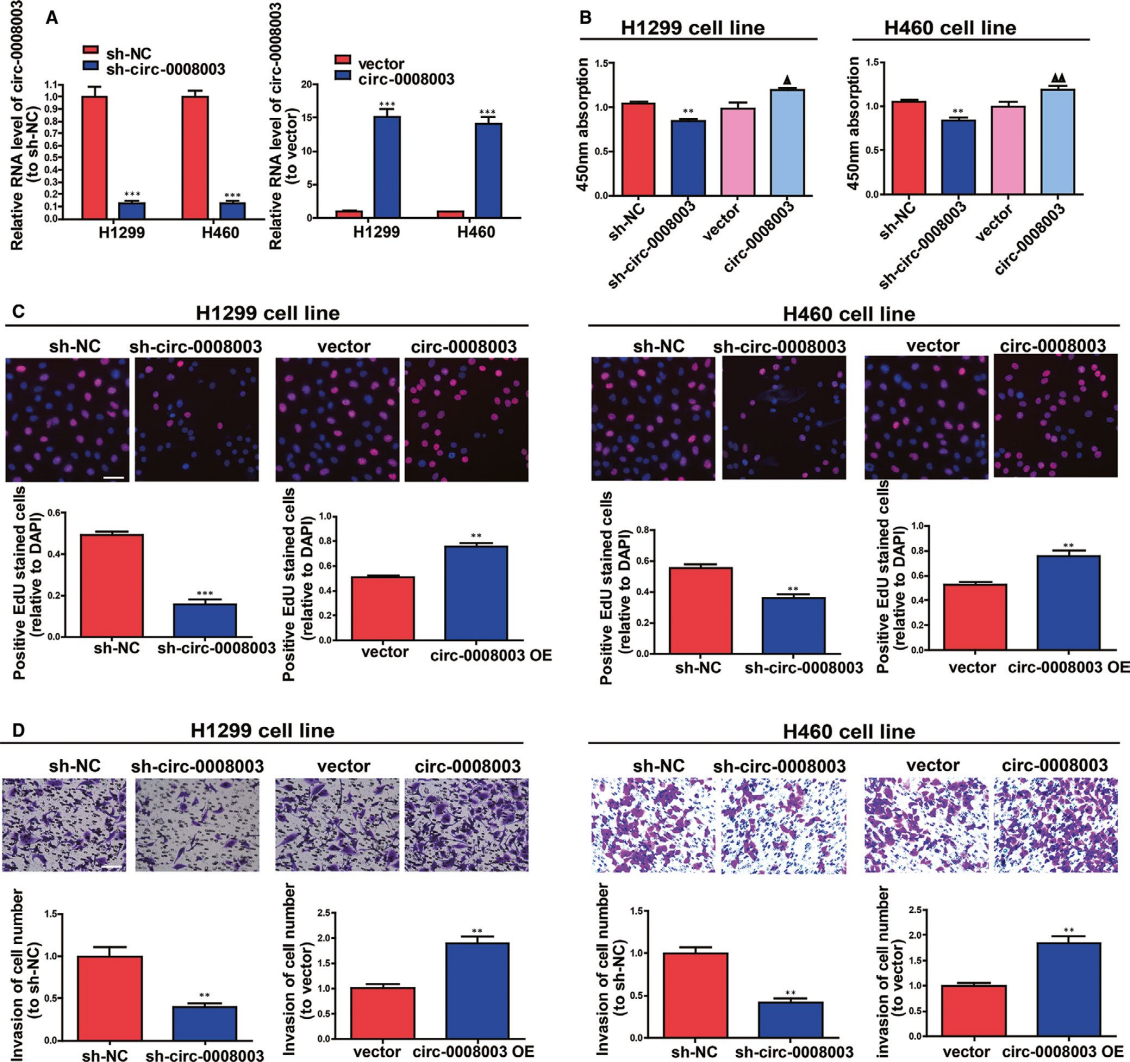
Effect of *circ‐0008003* on NSCLC cellular processes. A, Detection of *circ‐0008003* expression in H460 and H1299 cells after transfection via qRT‐PCR. Proliferation of H460 and H1299 cells following transfection are determined by CCK8 assay (B) or EDU assay (C), respectively (scale bar = 100 μm). D, The invasion abilities of H460 and H1299 cells after silencing or up‐regulating *circ‐0008003* are identified using Transwell assay (scale bar = 100 μm). Data are the mean ± SEM. **P < .01, ***P < .001

Because of the confirmed increase in *circ‐0008003* expression in NSCLC, its influences in NSCLC cell biological processes were tested by silencing or raising *circ‐0008003* expressions in H460 and H1299 cells. QRT‐PCR findings illustrated that *circ‐0008003* was remarkably pulled down or raised in NSCLC cells after the addition of *circ‐0008003* shRNA or overexpressing vector (Figure 2A). According to CCK8 and 5‐Ethynyl‐2'‐deoxyuridine (EDU) assays, the proliferation ability of NSCLC cells was decreased after silencing *circ‐0008003* and increased after up‐regulating *circ‐0008003* (Figure 2B‐C). Besides, the invasion abilities of the two cell lines became weak after silencing *circ‐0008003* and strong after up‐regulating *circ‐0008003* unfolded by Transwell assay (Figure 2D). These data reveal that *circ‐0008003* promotes NSCLC cell invasion and proliferation in vitro.

### 3.3 Tumour growth in vivo was slowed down by reduced *circ‐0008003* expression

**Figure 3:**
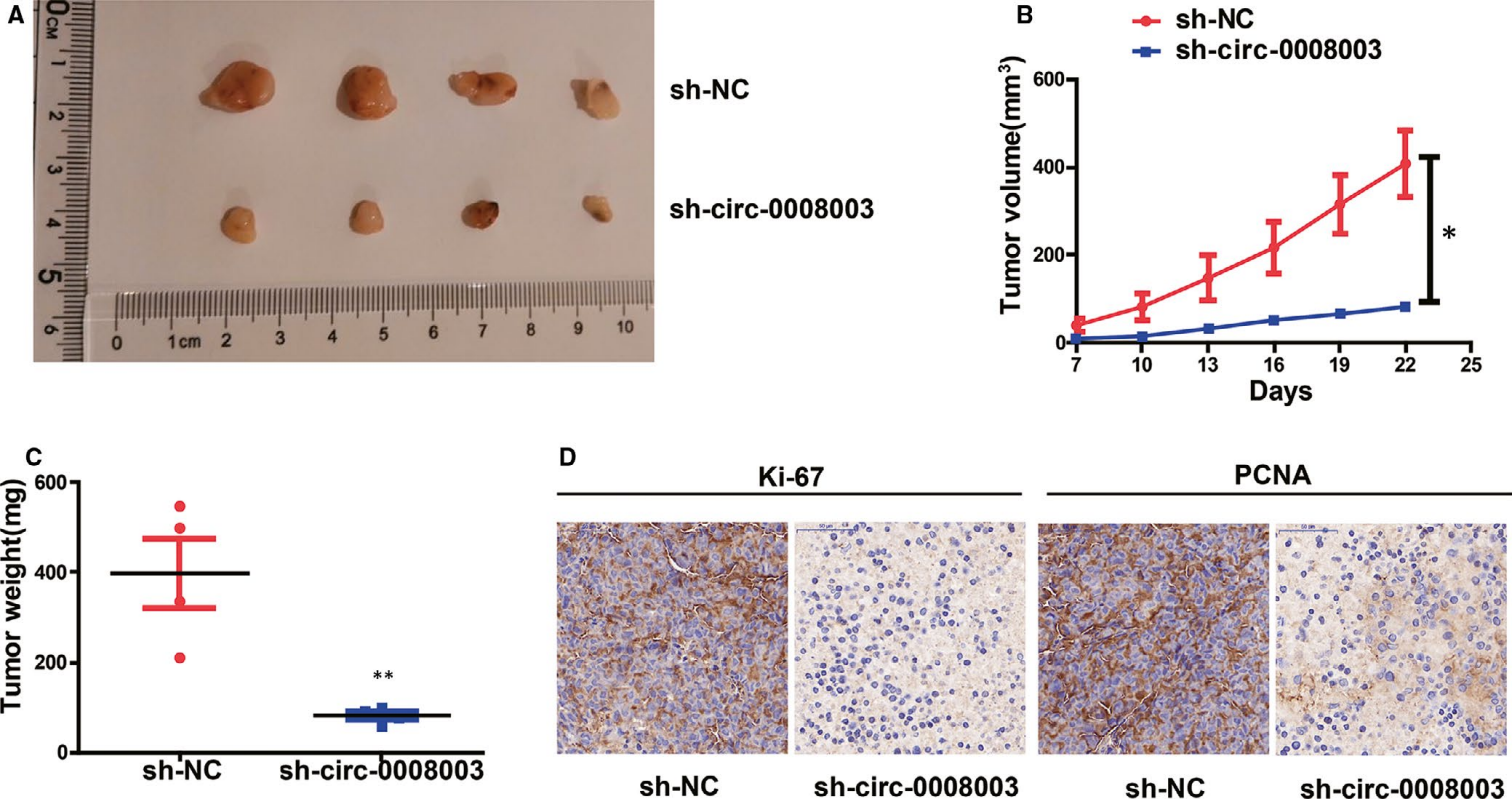
Inhibition of *circ‐0008003* impedes tumour growth in vivo. A, Typical xenograft images. B‐C, The tumour weight and size of these xenografts. D, Determination of Ki67 and PCNA expression in tumours from H460 cells after transfection via IHC staining (scale bar = 50 μm). Data are the mean ± SEM. *P < .05, **P < .01

H460 cells treated with *circ‐0008003* shRNA or NC were used for in vivo experiments to continuously prove the effect of *circ‐0008003* in tumour formation of NSCLC. In comparison with those in NC group, repressed *circ‐0008003* resulted in relatively small xenografts, identical to the results in vitro (Figure 3A). Moreover, relative to those in control group, tumours derived from H460 cells with repressed *circ‐0008003* had limited weight and size (Figure 3B,C). And IHC staining was adopted to examine the expressions of Ki67 and PCNA, the markers of cell proliferation.^26^, ^27^ Consequently, there were substantially less stained PCNA and Ki67 in tumours in *circ‐0008003*‐repressed cell group relative to those in NC‐treated H460 cell group (Figure 3D). It can be deduced that *circ‐0008003* repression inhibits tumour formation in vivo.

### 3.4 *Circ‐0008003* was considered to be an endogenous sponge for miR‐488 in NSCLC

**Figure 4:**
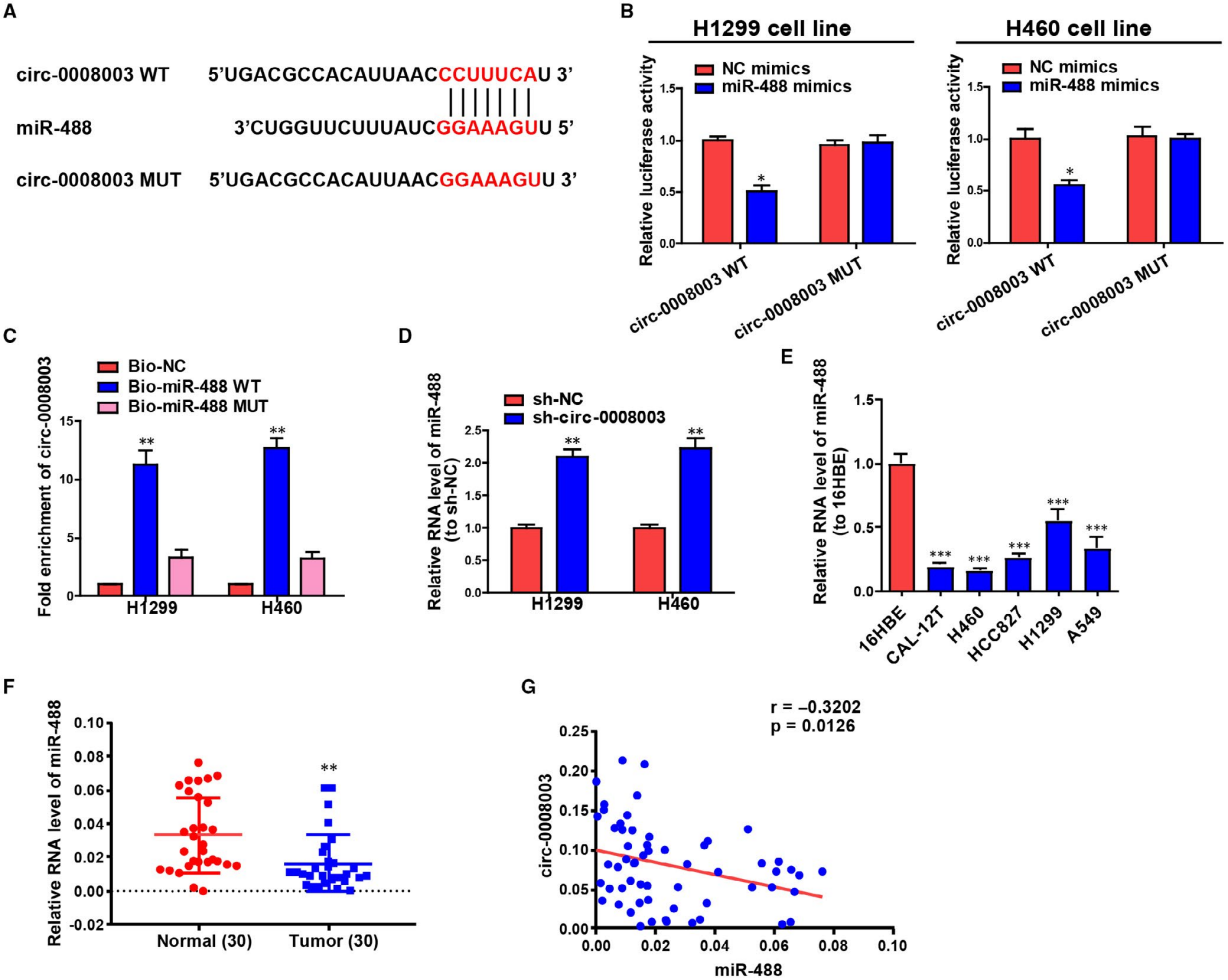
MiR‐488 is adjusted by *circ‐0008003* in NSCLC in a direct and negative manner. A, The forecasting binding sites between miR‐488 and *circ‐0008003* are obtained from Circinteractome. B, The relative luciferase activities of NSCLC cells treated with WT or MUT *circ‐0008003* reporter plasmids and miR‐488 mimics or NC mimics. C, RNA reduction experiment continuously verifies the interaction between miR‐488 and *circ‐0008003* in NSCLC cells. D, QRT‐PCR analysis of miR‐488 expression levels in H460 and H1299 cells following the inhibition of *circ‐0008003*. E, Measurement of miR‐488 expressions in NSCLC cell lines (CAL‐12T, H460, HCC827, H1299 and A549) and the human bronchial epithelioid cell line 16HBE via qRT‐PCR. F, Measurement of miR‐488 expressions in cancerous and para‐carcinoma tissues via qRT‐PCR. G, Association between *circ‐0008003* expression and miR‐488 in NSCLC tissues is estimated by Pearson's correlation analysis. Data are the mean ± SEM. *P < .05, **P < .01, ***P < .001

CircRNAs expressed in the cytoplasm have been demonstrated to substantially affect pathological and physiological processes by sponging miRNAs to some extent.^28^, ^29^ To ascertain the cellular localization of *circ‐0008003*, cells were isolated into the nucleus and cytoplasm fractions, in which the controls, U6 and GAPDH, could be seen. QRT‐PCR validated that *circ‐0008003* was monitored mainly in the cytoplasm fractions in NSCLC cells (Figure S1A). Identically, a substantial staining of *circ‐0008003* in the cytoplasm of H1299 cells was also determined in RNA‐FISH (Figure S1B). It can be seen that *circ‐0008003* probably functions in NSCLC at the post‐transcriptional level. To forecast *circ‐0008003* miRNAs with underlying interaction, the online bioinformatics tool Circinteractome was employed. As a result, *circ‐0008003* was unveiled to bind to miR‐488 with the highest potential, and the sequences of wild‐type *circ‐0008003* (with forecasted binding sites), mutant *circ‐0008003* (with mutant sites) and miR‐488 were displayed in Figure 4A. Subsequently, *circ‐0008003*'s interaction with miR‐488 primarily appeared in the cytoplasm rather than the nucleus of H460 and H1299 cells (Figure S1C,D), thus validating the above forecasting.

Then, luciferase reporter assay was conducted to ascertain the above‐mentioned interaction. It was illustrated that the luciferase activity of *circ‐0008003*‐WT could be repressed by miR‐488 mimics, but that of *circ‐0008003*‐MUT remained unchanged (Figure 4B). According to the RNA reduction experiment, *circ‐0008003* only gathered in complexes repressed not by Bio‐miR‐488‐Mut or Bio‐NC but by Bio‐miR‐488‐WT (Figure 4C). And repressing *circ‐0008003* expression led to the elevated level of miR‐488 in H460 and H1299 cells (Figure 4D). Further, miR‐488 expression in NSCLC cells and tissues was investigated through qRT‐PCR, the results of which ascertained that miR‐488 was markedly pulled down in NSCLC cells and tissues relative to the controls (Figure 4E,F). Eventually, the inverse relationship between the level of miR‐488 and *circ‐0008003* expression was uncovered by Pearson's correlation analysis in NSCLC patients (Figure 4G). These findings uncover that *circ‐0008003* may promote NSCLC progression through the inverse adjustment of miR‐488 expression.

### 3.5 Aberrant miR‐488 expression slowed down NSCLC progression

Figure S2A illustrated that the expression of miR‐488 was greatly inhibited or enhanced in NSCLC cells treated with miR‐488 inhibitor or mimics relative to that in cells treated with NC. Besides, miR‐488 impeded cell invasion and proliferation (Figure S2B‐D). Collectively, the results indicate that miR‐488 influences NSCLC evolution as a tumour suppressor.

### 3.6 *Circ‐0008003* accelerated NSCLC development by repressing miR‐488

**Figure 5:**
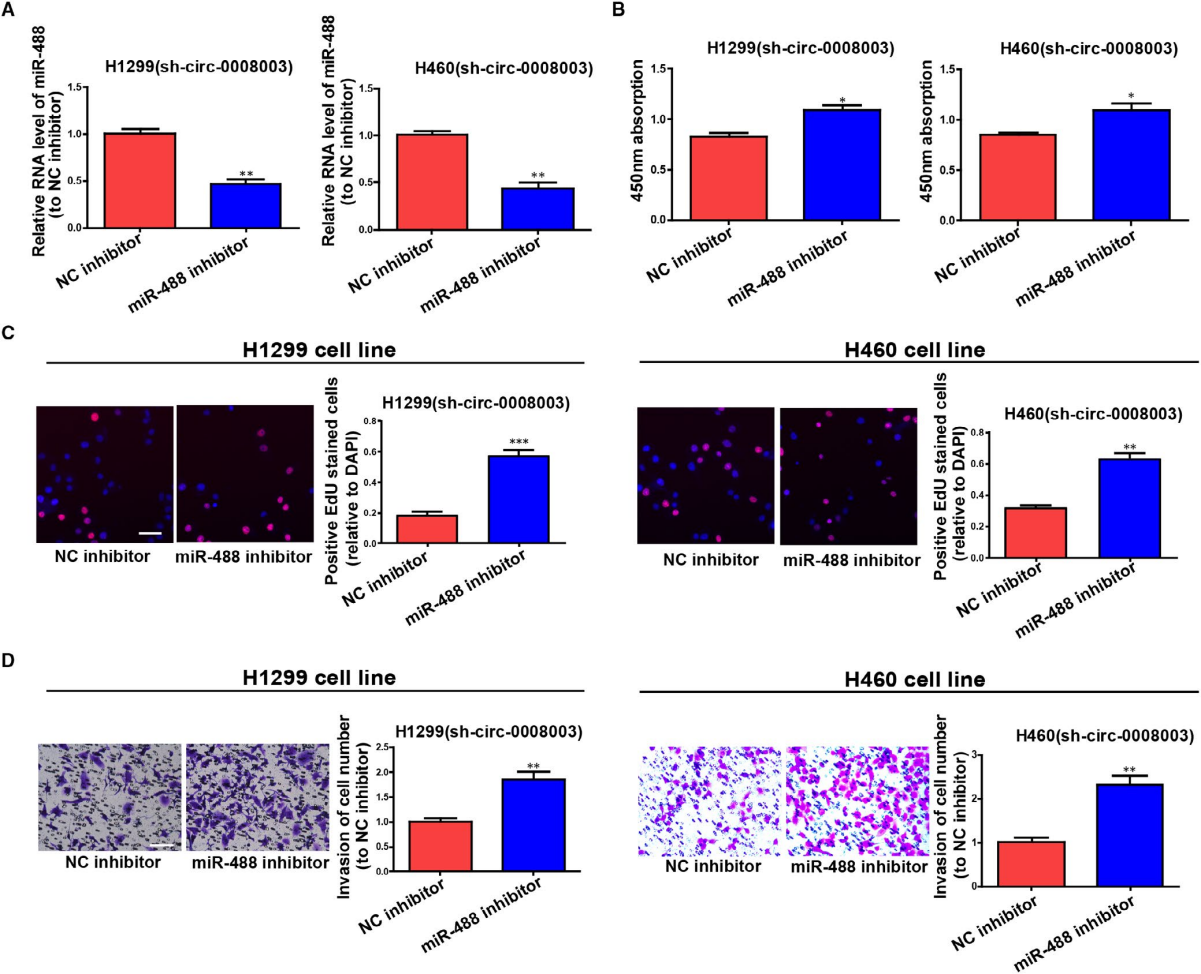
Effects of *circ‐0008003* inhibitor in NSCLC cells are reversed by silencing miR‐488 to some extent. A, Findings of miR‐488 relative expression in *circ‐0008003*‐repressed H460 and H1299 cells treated with miR‐488 inhibitor or NC inhibitor through qRT‐PCR. B‐C, Cell proliferation and (D) invasion capacities of *circ‐0008003*‐inhibited H460 and H1299 cells with or with no suppression of miR‐488 are analysed (scale bar = 100 μm). Data are the mean ± SEM. *P < .05, **P < .01, ***P < .001

To ascertain that the role of *circ‐0008003* required miR‐488 reduction in a direct manner, miR‐488 inhibitor was transfected into *circ‐0008003*‐repressed H460 and H1299 cells, and the observably reduced miR‐488 was discovered by qRT‐PCR (Figure 5A). A foregone conclusion was obtained from CCK8 and EDU assays that the proliferation capacities of *circ‐0008003*‐repressed H460 and H1299 cells were promoted by miR‐488 suppression (Figure 5B,C). Lastly, silencing miR‐488 was proved by Transwell assay to evidently boost the invasion capacities of *circ‐0008003*‐repressed H460 and H1299 cells (Figure 5D). The above discovery implies that miR‐488 participates in the carcinogenic process regulated by *circ‐0008003* in NSCLC cells.

### 3.7 *ZNF281* was a direct target of miR‐488

**Figure 6:**
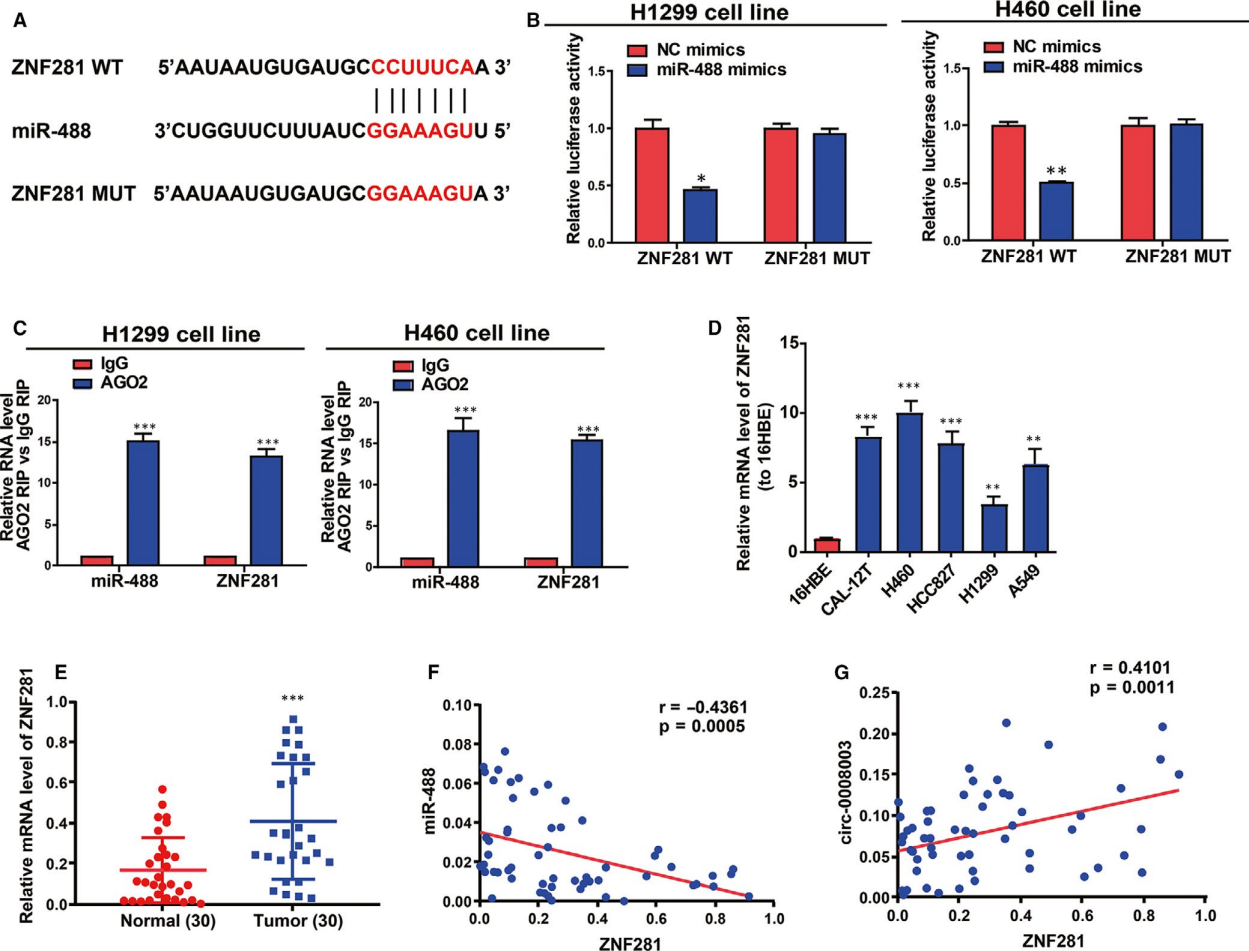
*ZNF281* is the direct target of miR‐488. A, The binding sites between miR‐488 and *ZNF281* 3'UTR are forecasted by bioinformatics prediction. B, Luciferase reporter assay shows the specific forecasting binding sites between miR‐488 and *ZNF281* in NSCLC cells. C, Interaction between miR‐488 and *ZNF281* mRNA in H460 and H1299 cells is confirmed by RIP assay. D, Measurement of *ZNF281* expressions in NSCLC cell lines (CAL‐12T, H460, HCC827, H1299 and A549) and the human bronchial epithelioid cell line 16HBE via qRT‐PCR. E, *ZNF281* expressions in 30 NSCLC tissues and matched para‐carcinoma tissues are measured using qRT‐PCR. F‐G, Pearson's correlation analysis is employed to figure out the correlation between *ZNF281* expression and the level of miR‐488 or *circ‐0008003* in 30 NSCLC tissues. Data are the mean ± SEM. *P < .05, **P < .01, ***P < .001

Next, the specific potential mechanism of *circ‐0008003*/miR‐488 axis in stimulating NSCLC evolution was discussed. With bioinformatics prediction tools (TargetScan, miRDB and Starbase), *ZNF281*, a carcinogene in cancers, was found to be a target of miR‐488 (Figure 6A). Besides, the direct binding of miR‐488 to *ZNF281* 3'UTR at the forecasting sites was ascertained in NSCLC cells (Figure 6B). From immunoprecipitate conjugated with Ago2 in H460 and H1299 cells, miR‐488 and *ZNF281* mRNA were obtained, validating the existence of the interaction between miR‐488 and *ZNF281* mRNA in RNA‐induced silencing complex (RISC) adjusted by miR‐488 (Figure 6C). Next, the expression of *ZNF281* was disclosed to notably rise in NSCLC cells and tissues relative to controls (Figure 6D‐E). What's interesting was that miR‐488 level was shown as an inverse correlator while *circ‐0008003* expression as a positive correlator of *ZNF281* expression in NSCLC tissues (Figure 6F,G). As a result, *ZNF281* may affect *circ‐0008003*/miR‐488 axis in NSCLC evolution.

### 3.8 *ZNF281* positively adjusted *circ‐0008003* in NSCLC evolution

**Figure 7:**
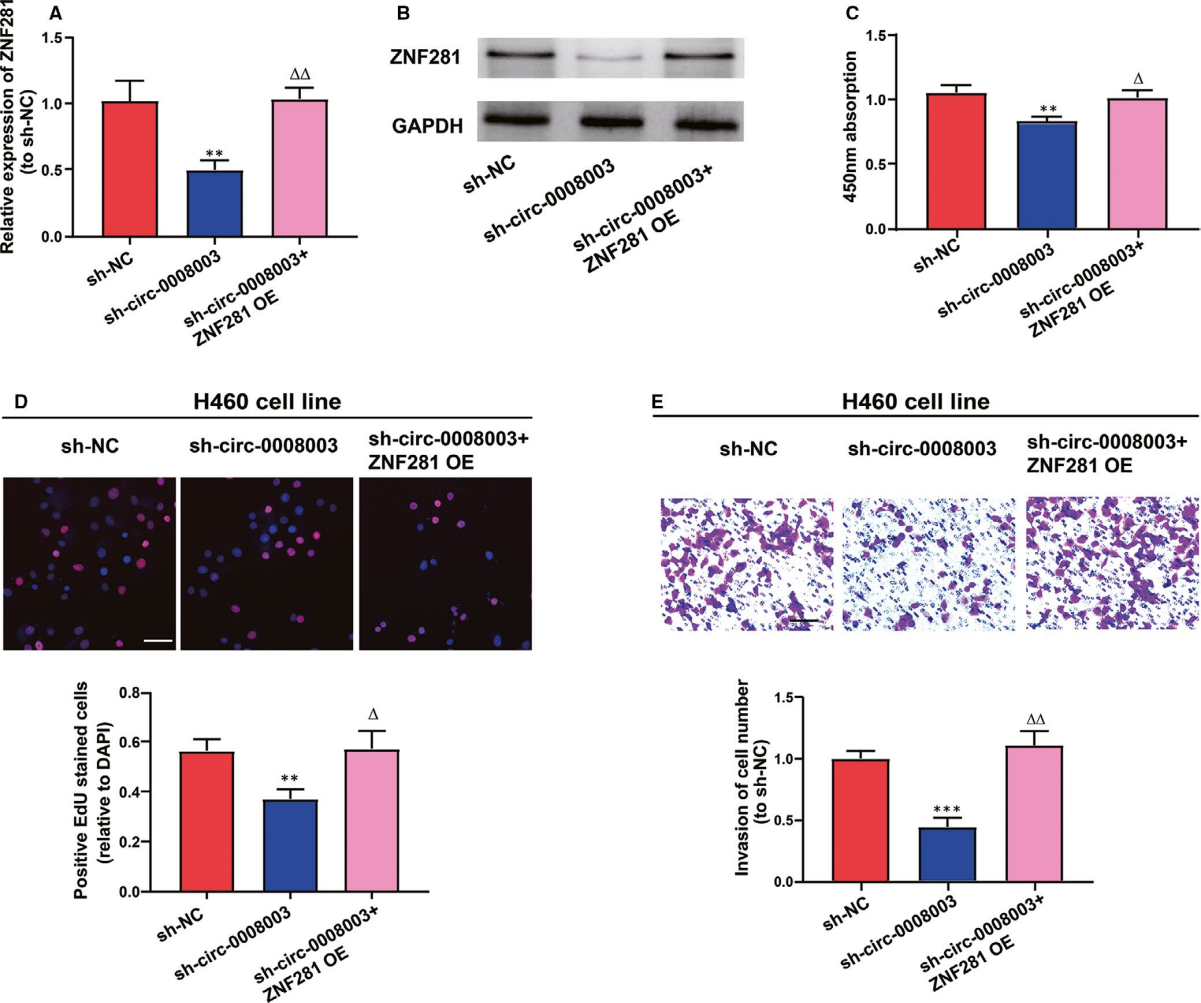
*ZNF281* overexpression restores the repression of *circ‐0008003* inhibition in NSCLC. A‐B, The mRNA and protein levels of *ZNF281* in H460 cells with NC shRNA, *circ‐0008003* shRNA, or together with *ZNF281* elevation, are assessed using qRT‐PCR and Western blotting, respectively. C‐D, CCK8 and EDU assays are carried out to ascertain the proliferation capacity of H460 cells under different conditions (scale bar = 100 μm). E, Invasion ability of H460 cells receiving various treatments is tested through Transwell assay (scale bar = 100 μm). Data are the mean ± SEM. **P < .01, ***P < .001 vs control group, ^△^ *P* < .05, ^△△^ *P* < .01 vs circ‐0008003 shRNA group

To figure out the adjustment of *ZNF281* on the role of *circ‐0008003* in NSCLC evolution, the function of *ZNF281* overexpression in the biological processes of *circ‐0008003*‐repressed H460 cells was analysed. In the first place, it was revealed that mRNA and protein expression levels of *ZNF281* were remarkably impeded after the repression of *circ‐0008003* and became normal after cotransfection with *ZNF281* overexpression vector (Figure 7A,B). Afterwards, *circ‐0008003*‐repressed H460 cells predominantly regained their repressed proliferation abilities following cotransfection with *ZNF281* overexpression vector (Figure 7C,D). Additionally, *ZNF281* overexpression alleviated the hampering of *circ‐0008003*‐repressed invasion processes in H460 cells (Figure 7E). Basically, *circ‐0008003* facilitates NSCLC evolution via *circ‐0008003*/miR‐488/*ZNF281* axis.

## 4 DISCUSSION

CircRNAs have been demonstrated to substantially influence different biological processes in cells.^30^, ^31^ According to reports, aberrant adjustment of circRNAs exerts effects on cancer evolution and is associated with patient's prognosis.^32^, ^33^ CircRNAs have been recently proved to pivotally affect NSCLC.^34^ For example, circRNA *hsa_circ_0008305* (*circPTK2*) impedes EMT and metastasis triggered by TGF‐β through controlling TIF1γ in NSCLC^35^; circRNA *F‐circEA‐2a* originated from EML4‐ALK fusion gene boosts invasion and migration of NSCLC cells.^36^ In this research, elevation of *circ‐0008003* in NSCLC tissues and cells was discovered, and high level of *circ‐0008003* had a positive relationship to poor prognosis, TNM stage and lymphatic metastasis. Moreover, repression of *circ‐0008003* hampered tumour growth in vivo as well as the cell invasion and proliferation in vitro. The findings validate that *circ‐0008003* affects NSCLC development and progression like an oncogene.

Verified by multiple reports, circRNAs adjust different cancers through targeting mRNAs or miRNAs.^37^, ^38^ CircRNA *hsa_circ_0091570* blocks HCC progression by sponging hsa‐miR‐1307 like a ceRNA.^20^ CircRNA *hsa_circRNA_100290* adjusts cell growth in oral squamous cell carcinoma by GLUT1 and glycolysis like a ceRNA for miR‐378a.^39^ CircRNA *CircCACTIN* accelerates the evolution of gastric cancer by sponging miR‐331‐3p and mediating the expression of TGFBR1.^40^ Also, circRNAs serve as an endogenous sponge for miRNAs in NSCLC. For instance, circRNA 100146 exerts an oncogenic effect by binding to miR‐615‐5p and miR‐361‐3p in NSCLC in a direct manner.^41^ Currently, it was uncovered that *circ‐0008003* with expression primarily in the cytoplasm accelerates NSCLC evolution by sponging miR‐488 that represses NSCLC,^42^, ^43^ which was identified as a suppressor gene in NSCLC in this research.

*ZNF281* is located at chromosome 1q32.1 and is a critical regulator of embryonic stem cell differentiation and tissue development.^44^ Multiple studies have demonstrated the diverse roles of *ZNF281* in stem cell pluripotency and development processes.^45^, ^46^ Therefore, *ZNF281* is regarded as a relevant gene for cellular stemness. Notably, recent investigations have revealed that *ZNF281* is a novel oncoprotein that is frequently overexpressed in human malignancies, including colorectal and pancreatic cancers.^47^, ^48^ More importantly, high *ZNF281* expression in cancer tissues enhances metastatic potential and reduces patient survival.^46^, ^49^, ^50^ However, its actual role and underlying mechanism in NSCLC have not yet been elucidated. This research showed that *ZNF281* was a target of miR‐488 and *circ‐0008003* affected NSCLC via adjusting miR‐488/*ZNF281* signalling like an oncogene.

To sum up, *circ‐0008003* plays a carcinogenic role in NSCLC development via sponging miR‐488, thus releasing and increasing *ZNF281*. This research firstly evidences circ‐0008003's identities as an underlying treatment marker and a prognostic target in NSCLC, but its clinical significance still needs in‐depth investigations in the future.

## CONFLICT OF INTEREST

The authors state that they have no conflict of interest.

## AUTHOR CONTRIBUTIONS

**Runhuan Gu:** Investigation (equal); Writing‐original draft (equal). **Koufeng Shao:** Methodology (equal); Validation (equal). **Qiaoxia Xu:** Data curation (equal); Software (equal); Visualization (equal). **Xue Zhao:** Formal analysis (equal); Software (equal); Visualization (equal). **Haibing Qiu:** Project administration (equal); Resources (equal); Writing‐review & editing (equal). **Haibo Hu:** Conceptualization (lead); Resources (lead).

## Supporting information

Fig S1

Fig S2

Supplementary Material

## Data Availability

The data that support the findings of this study are available from the corresponding author upon reasonable request.
