# Supplementary figures and images for: Circular RNA hsa_circ_0008003 facilitates tumorigenesis and development of non‐small cell lung carcinoma via modulating miR‐488/ZNF281 axis
Source: J Cell Mol Med. 2020 Dec 15;26(6):1754–65. doi: 10.1111/jcmm.15987 (PMC8918407; doi:10.1111/jcmm.15987)

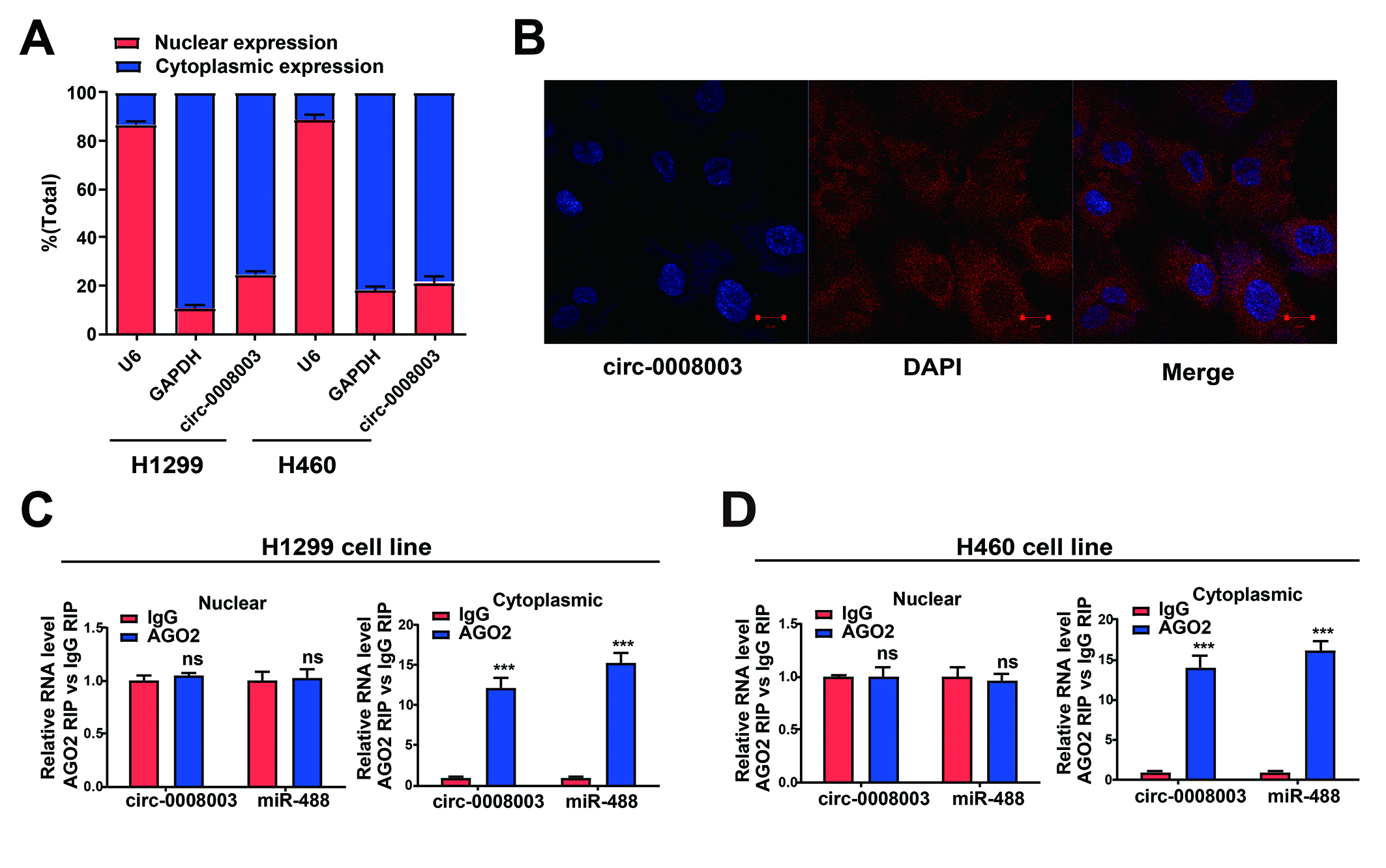

Supplement: Supplementary file 1 — Fig S1 [file JCMM-26-1754-s003.tif]

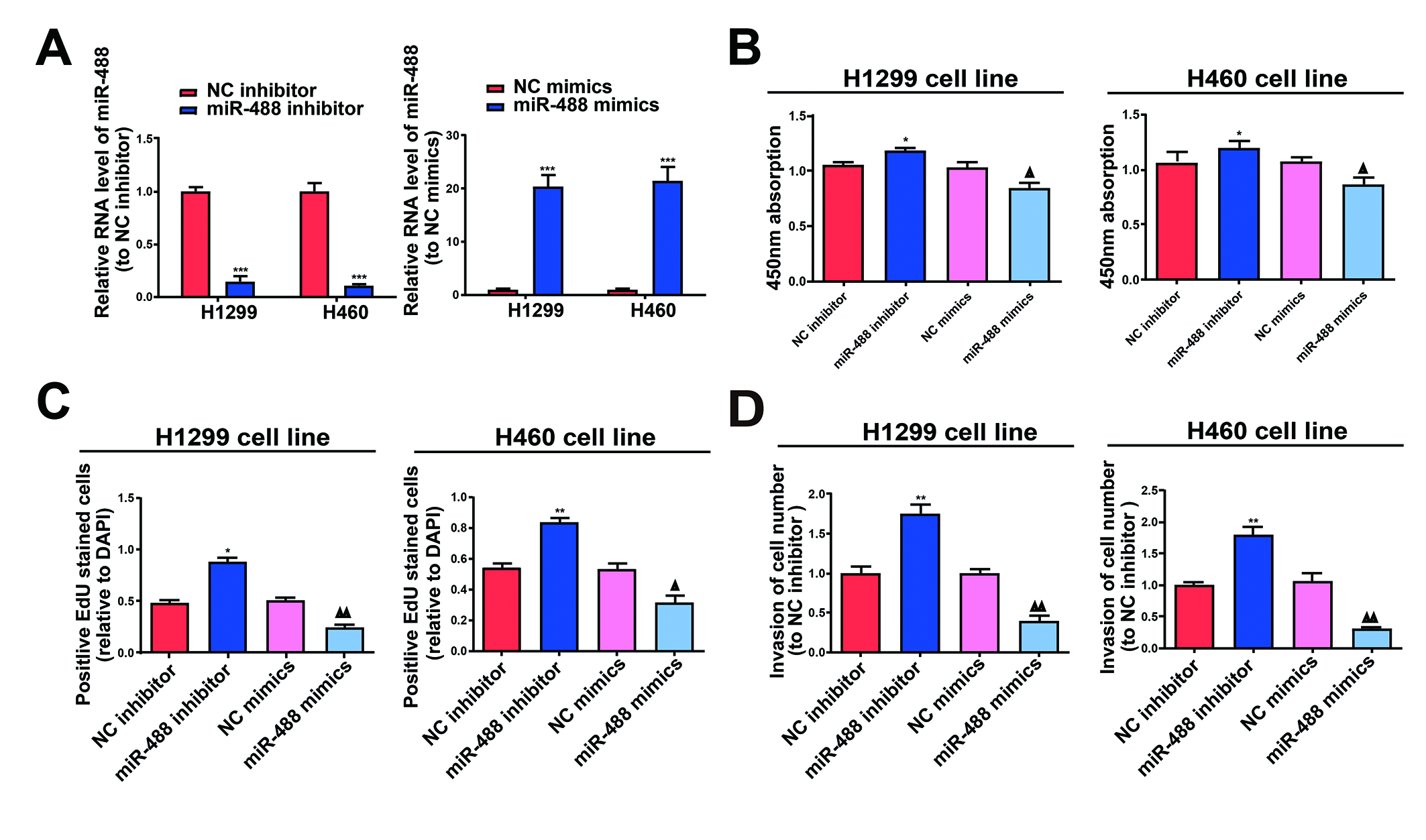

Supplement: Supplementary file 2 — Fig S2 [file JCMM-26-1754-s001.tif]
