# Supplementary material for: Circular RNA hsa_circ_0008003 facilitates tumorigenesis and development of non‐small cell lung carcinoma via modulating miR‐488/ZNF281 axis
Source: J Cell Mol Med. 2020 Dec 15;26(6):1754–65. doi: 10.1111/jcmm.15987 (PMC8918407; doi:10.1111/jcmm.15987)
Supplement: Supplementary file 3 — Supplementary Material [file JCMM-26-1754-s002.docx]

**Supplementary Figure legends**

**Figure S1. Subcellular localization of circ-0008003.** (A) Circ-0008003 was detected mainly in the cytoplasm fractions in NSCLC cells. (B) RNA-FISH showed a dramatic cytoplasmic staining of circ-0008003 in H1299 cells (scale bar=20μm). (C-D) RIP assay showed the interactivity of circ-0008003 with miR-488 mainly occurred in the cytoplasm of H1299 cells (C) and H460 cells (D) while that in the nucleus happened scarcely. Data are the mean ± SEM. *P<0.05, ** P<0.01, ***P< 0.001, ns means no significance.

**Figure S2. Ectopic expression of miR-488 led to repressive impact on NSCLC progression.** (A) The expressions of miR-488 in NSCLC cells after transfection. (B-C) miR-488 showed a suppressive effect on cell proliferation. (D) miR-488 showed a suppressive effect on cell invasion. Data are the mean ± SEM. *P<0.05, ** P<0.01, ***P< 0.001. *P <0.05 vs. NC inhibitor, ^△^P <0.05 vs. NC mimics.
